# Genetic and nongenetic variation of multiple births in Austrian dual-purpose cows

**DOI:** 10.3168/jdsc.2024-0576

**Published:** 2024-07-14

**Authors:** M. Caccin, B. Fuerst-Waltl, C. Fuerst, A. Costa, M. Penasa

**Affiliations:** 1 Department of Agronomy, Food, Natural Resources, Animals and Environment, University of Padova, 35020 Legnaro (PD), Italy; 2 Institute of Livestock Sciences, BOKU University, Vienna, 1180 Vienna, Austria; 3 ZuchtData EDV-Dienstleistungen GmbH, 1200 Vienna, Austria; 4 Department of Veterinary Medical Sciences, Alma Mater Studiorum, University of Bologna, 40064 Ozzano dell'Emilia (BO), Italy

## Abstract

In recent decades, a general increase in multiple birth (MB) rate has been reported in both dairy and dual-purpose cattle breeds. As there is evidence that MB has negative effects on economically important traits in dairy cows, the aims of this study were to (i) investigate environmental and genetic factors affecting MB rate and (ii) assess the impact of MB on productive and nonproductive traits of the Austrian dual-purpose breeds Pinzgauer and Tyrol Grey. The dataset included 99,141 calvings of 33,791 Pinzgauer and 68,454 calvings of 19,244 Tyrol Grey cows recorded from 2000 to 2022. Environmental factors affecting MB were investigated using logistic regression, and genetic parameters of MB were estimated using linear and threshold animal models. In addition, the effects of MB on milk, fat, and protein yields and calving ease were analyzed using a linear mixed model, whereas stillbirth and cow survival (binary traits) were analyzed by logistic regression. The average MB rate from 2000 to 2022 was 5.80% for Pinzgauer and 3.89% for Tyrol Grey. The lowest MB rate was observed in primiparous cows in both breeds, and the highest in fourth-parity Pinzgauer and seventh-parity Tyrol Grey cows. The highest MB rate was in summer regardless of the breed. In Pinzgauer, the MB negatively affected dairy traits and survival, and in Tyrol Grey, it affected fat yield and calving ease. Stillbirth was notably higher when MB occurred than in single births in both breeds. In Pinzgauer, the estimated heritability was 0.029 (linear) and 0.138 (threshold), whereas in Tyrol Grey, it was 0.040 (linear) and 0.200 (threshold). Low additive genetic variation exists for this trait, but it is still exploitable in genetic programs aimed at curbing the MB rate.

## Highlights

• MB was investigated in Pinzgauer and Tyrol Grey cattle.
• The highest MB rate in these dual-purpose breeds was recorded in summer.
• First-parity cows had the lowest MB rate.
• MB negatively affected milk production, calving traits, and cow survival.
• Exploitable additive genetic variation exists to select against or stabilize MB.

## Graphical Abstract

**Figure:**
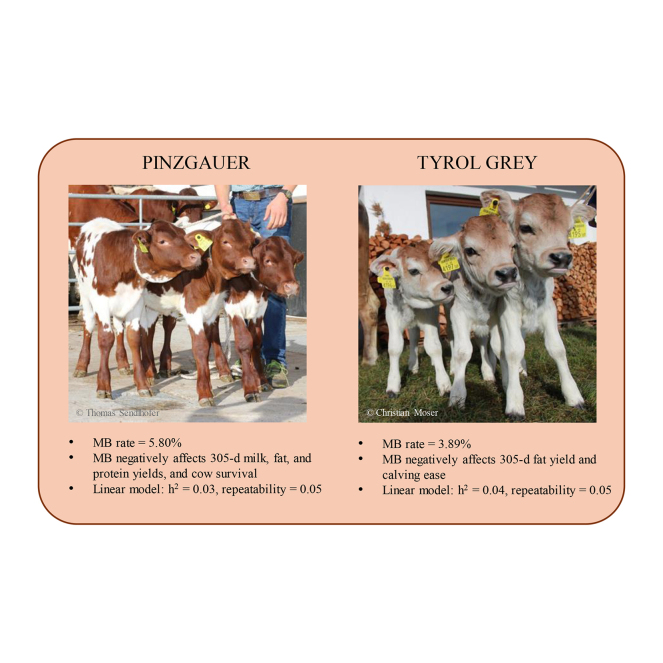
**Summary:** Multiple births (MB) are undesirable in dairy and dual-purpose cattle herds. This study aimed to investigate the factors affecting MB and assess its impact on traits of economic relevance in dual-purpose Pinzgauer and Tyrol Grey cattle breeds in Austria. The highest MB rate was recorded in summer. First-parity cows had the lowest MB rate, and the greatest difference was reported between first and second parity. Overall, MB had negative effects on milk yield traits, calving traits, and cow survival. Additive genetic variation exists for MB in the studied breeds, meaning that there is room to reduce or stabilize its incidence through tailored selection.

Multiple birth (**MB**) rates are generally higher in dairy and dual-purpose breeds than in beef cattle breeds, and their incidence is increasing (Cabrera and Fricke, 2021). Rutledge (1975) estimated a maximum MB rate of 1.0% in beef cattle, Cady and Van Vleck (1978) reported an MB rate of 4.75% in Holstein cattle from the Midwestern United States, and Atteneder (2007) reported MB rates of 5.57% and 3.92% for Pinzgauer and Tyrol Grey, respectively.

In general, MB is somewhat desirable for beef cattle in some contexts, as it results in a higher direct profit for the farmer. In specialized dairy cattle breeds, this apparent advantage does not pay off because MB may result in severe negative effects on both dams and newborns. Multiple birth is generally associated with an increased demand for calving assistance (Andreu-Vázquez et al., 2012), along with a higher risk of placental retention, metritis or endometritis (or both), dystocia, displaced abomasum, prolonged days open, and poorer postcalving BCS (Beerepoot et al., 1992; Bell and Roberts, 2007). Moreover, MB is associated with a higher incidence of abortion and stillbirth, shorter gestation length, and lighter birth weight (López-Gatius et al., 2004; Hossein-Zadeh et al., 2008), along with freemartinism. All the negative effects that may arise from MB lead to economic losses for the farmer (Cabrera and Fricke, 2021). Fodor et al. (2019) reported a loss of $59 per twinning case in Hungarian Holstein-Friesian herds and Mur-Novales et al. (2018) estimated losses from $97 to $225 per twin pregnancy under different scenarios. Clearly, 2 female twins can be considered 2 potential replacement heifers in dairy herds. However, it is important to emphasize that, in local and dual-purpose breeds, with little or no interest in sexed semen, the probability of obtaining 2 females is only 25%, disregarding monozygotic twins, which are rare.

The occurrence of MB depends on both environmental and cow-related factors. Among the former, season plays an important role, with autumn having the highest frequency of multiple ovulations and thus summer having the highest incidence of MB events (Sawa et al., 2015). Cow-related factors primarily include age/parity of the dam and milk production, with a higher MB rate in multiparous than primiparous cows (Komisarek and Dorynek, 2002), and more productive than less productive cows (López-Gatius et al., 2005). Genetics is another factor that plays a role in MB occurrence. Several QTLs, located on different chromosomes, are associated with high ovulation and twinning rates (Komisarek and Dorynek, 2002; Widmer et al., 2022). In terms of polygenic evidence, heritability (h^2^) and repeatability (t) of MB have been reported to be low in cattle, and estimates mainly vary depending on breed, parity, accuracy of the phenotypes available, and statistical approach (Moioli et al., 2017; Lett and Kirkpatrick, 2018).

Given the negative impact that MB can have in the herd, the present study aimed to (i) investigate environmental and genetic factors affecting MB rate and (ii) assess the impact of MB on productive and nonproductive traits of 2 Austrian dual-purpose cattle breeds.

Data of dual-purpose Pinzgauer and Tyrol Grey cows spanning the years 2000 to 2022 were provided by ZuchtData EDV-Dienstleistungen GmbH (Vienna, Austria). Only herds with at least 50 calvings for Pinzgauer and 30 calvings for Tyrol Grey, and calvings with known birth type (single, twin, triple, or higher births) were retained. In addition, cows of parity greater than 15 and/or with a proportion of genes from other breeds greater than 6.25% due to non-purebred ancestors were discarded.

After editing, the dataset included information on 99,141 calvings from 33,791 Pinzgauer cows in 646 herds and 68,454 calvings from 19,244 Tyrol Grey cows in 608 herds. In addition to birth type, the following traits were considered: (i) stillbirth, defined as the presence or absence of at least one calf stillborn or dead within 48 h after parturition for each calving; (ii) calving ease, evaluated on a scale from 1 to 5, where 1 is unassisted calving and 5 is embryotomy; (iii) cow survival, defined as cow culled or dead within the first 150 d after calving; and (iv) 305-d milk, fat, and protein yield (kg), for lactations ≥250 d. Birth type was simplified to a binary trait, whereby 0 corresponded to single birth and 100 to MB (twin, triple, and higher births), and calving ease scores were reduced to 3 categories: 1 = easy calving, 2 = calving assisted by one person, and 3 = difficult calving, which included the original categories 3, 4, and 5.

A 6-generation pedigree was provided by ZuchtData EDV-Dienstleistungen GmbH (Vienna, Austria) for the animals in the original dataset. The RelaX2 program (Stranden and Vuori, 2006) was used to remove animals that did not contribute information to the estimation of variance components and to trace the pedigree separately for the final datasets of the 2 breeds. This resulted in 60,552 and 27,429 animals for Pinzgauer and Tyrol Grey, respectively.

Birth type was analyzed using logistic regression with binary distribution and logit link function using the GLIMMIX procedure of SAS software v.9.4 (SAS Institute Inc., Cary, NC). The model was as follows:

[1] *y_ijklmn_* = *μ* + *parity_i_* + *year_j_* + *season_k_* + *herd_l_* + *cow_m_*(*herd_l_*) + *e_ijklmn_*,

where *y_ijklmn_* is birth type (0 or 100); *μ* is the overall intercept of the model; *parity_i_* is the fixed effect of the *i*th parity of the cow (*i* = 1, 2, 3, 4, 5, 6, 7, ≥8); *year_j_* is the fixed effect of the *j*th year of calving (*j* = 2000 to 2022); *season_k_* is the fixed effect of the *k*th season of calving (*k* = winter, January to March; spring, April to June; summer, July to September; autumn, October to December); *herd_l_* is the random effect of the *l*th herd (*l* = 1 to 646 for Pinzgauer, and 1 to 608 for Tyrol Grey); *cow_m_*(*herd_l_*) is the random effect of the *m*th cow nested within the *l*th herd (*m* = 1 to 33,791 for Pinzgauer, and 1 to 19,244 for Tyrol Grey); and *e_ijklmn_* is the random residual. Results are presented as back-transformed LSM of birth type for the fixed effects, and Bonferroni adjustment was adopted for multiple comparisons (*P* < 0.05).

The effect of birth type on 305-d milk, fat, and protein yield and calving ease was investigated using a linear mixed model with normal distribution and identity link function, and the effects of birth type on stillbirth and cow survival were analyzed using a logistic regression model with binary distribution and logit link function. The fixed and random factors considered in the models were the same as those reported for model [1], with the addition of the fixed effect of the birth type. Results for stillbirth and cow survival for the fixed effect of birth type are presented as back-transformed LSM. The analyses were run separately within each breed using the GLIMMIX procedure of SAS.

Variance components of birth type were estimated within the breeds using both a linear and a threshold animal model in ASReml software v.4 (Gilmour et al., 2015). Preference is generally given to linear models for routine genetic and genomic evaluations worldwide, as it has been shown to be robust to deviations from normality and perform as well as theoretically more appropriate threshold models (Weller et al., 1988, Koeck et al., 2010). In fact, binary traits are often included in multivariate analyses with other continuous traits. The linear model in matrix notation was as follows:

[2] **y** = **Xβ** + **Z_h_h** + **Z_pe_pe** + **Z_a_a** + **e**,

where **y** is a vector of observations of birth type; **β** is a vector of systematic fixed effects (i.e., parity [5 classes, from 1 to ≥5], year-season of calving [92 classes], and herd [646 for Pinzgauer and 608 for Tyrol Grey]); **h** is a vector of random herd-year effects; **pe** is a vector of random permanent environmental effects; **a** is a vector of random animal additive genetic effects; **e** is a vector of random residuals; and **X**, **Z_h_**, **Z_pe_**, and **Z_a_** are known incidence matrices relating birth type records to **β**, **h**, **pe**, and **a**, respectively. The h^2^ and t of MB were calculated as follows:

$$h^{2}=\frac{\sigma_{a}^{2}}{\sigma_{a}^{2}+\sigma_{pe}^{2}+\sigma_{h}^{2}+\sigma_{e}^{2}}$$

$$t=\frac{\sigma_{a}^{2}+\sigma_{pe}^{2}}{\sigma_{a}^{2}+\sigma_{pe}^{2}+\sigma_{h}^{2}+\sigma_{e}^{2}},$$

where $\sigma_{a}^{2}$ is the additive genetic variance; $\sigma_{pe}^{2}$ is the permanent environmental variance; $\sigma_{h}^{2}$ is the herd-year variance; and $\;\sigma_{e}^{2}$ is the residual variance.

The same fixed and random effects were used in the threshold model. A logit link function was adopted for the threshold model, which holds the residual variance to π^2^/3 in ASReml (Gilmour et al., 2015), and then h^2^ on liability scale $\left(h_{l}^{2}\right)$ was converted to observed (i.e., linear) h^2^ $\left(h_{o}^{2}\right)$ by adapting the formula of Dempster and Lerner (1950):

$$h_{o}^{2}=\frac{h_{l}^{2}\times z^{2}}{p\;\times \;\left(1-p\right)},$$

where *z*^2^ is the ordinate under the standardized normal distribution corresponding to *p*, and *p* is the prevalence of MB in the population. The same formula was adopted for the conversion of the SE.

Spearman's rank correlation coefficients were calculated between EBV predicted using linear and threshold models for bulls with an accuracy ≥0.70 (i.e., 213 and 226 bulls for Pinzgauer and Tyrol Grey, respectively).

The average MB rate was 5.80% for Pinzgauer (5.76% twins and 0.04% triplets/quadruplets) and 3.89% for Tyrol Grey (3.87% twins and 0.02% triplets). Similar rates for these breeds were reported by Atteneder (2007): 5.61% for Pinzgauer (5.57% twins and 0.04% triplets/quadruplets) and 3.94% for Tyrol Grey (3.92% twins and 0.02% triplets/quadruplets). The average twinning rate in US Holsteins was intermediate to that of the 2 dual-purpose breeds (4.80%; Lett and Kirkpatrick, 2018).

Parity and calving season effects were significantly associated with birth type in both Pinzgauer and Tyrol Grey, whereas calving year was significantly associated with birth type in Tyrol Grey only. The lowest MB rate was observed in primiparous cows (2.71% for Pinzgauer and 1.09% for Tyrol Grey) and the highest in multiparous cows (*P* < 0.05), with values ranging from 6.14% (parity 2) to 7.47% (parity 4) in Pinzgauer, and 3.59% (parity 2) to 4.71% (parity 7) in Tyrol Grey (Figure 1). Hossein-Zadeh et al. (2008) recorded a twinning rate of 1.1%, 4.2%, and 5.7% in first-, second-, and third-parity Iranian Holsteins. In US Holsteins, Lett and Kirkpatrick (2018) estimated a twinning rate of 0.30% for first-parity, 4.25% for second-parity, 6.90% for third-parity, and 7.63% for ≥fourth-parity cows. Multiple birth rate was erratic across years of calving, with the highest and lowest values in 2008 (7.03%) and 2001 (4.81%) for Pinzgauer (*P* < 0.05) and in 2000 (4.93%) and 2022 (3.19%) for Tyrol Grey (*P* = 0.42). These results agree with findings reported for other Austrian dairy cattle breeds, except for Fleckvieh whose trend increased across years, partly due to an extended length of productive life, which, in turn, resulted in a greater incidence of twin calvings within the population (C. Fuerst, ZuchtData, Vienna, Austria, unpublished data). Silva del Río et al. (2007) observed an increase of MB rate for Holstein cows in Minnesota from 1996 (3.4%) to 2004 (4.8%). The MB rate was higher in summer than in the other seasons (*P* < 0.05), with values of 7.46% for Pinzgauer and 5.10% for Tyrol Grey (Figure 1). Nielen et al. (1989) reported an increase in twin births from April to September in Dutch Friesians and in crosses between Dutch and Holstein Friesians, and Lett and Kirkpatrick (2018) observed that summer had the highest MB rate (5.19%) in US Holsteins. De Rensis and Scaramuzzi (2003) argued that in the Northern Hemisphere, cows experiencing heat stress during summer tend to have a single persistent dominant preovulatory follicle. Follicular dominance and persistence decrease after summer, making multiple conceptions highly possible in autumn and winter and thus more MB in the following summer.

**Figure 1:**
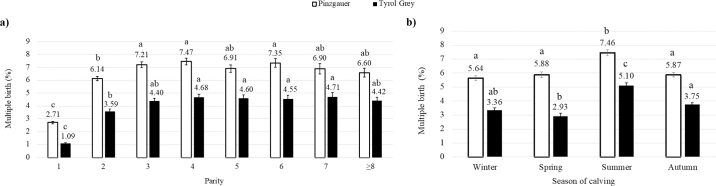
Back-transformed LSM of multiple birth rate for the fixed effects of (a) parity and (b) season of calving. Means with different letters (a–c) within breed differ significantly (*P* < 0.05).

Birth type significantly affected 305-d milk, protein, and fat yields, stillbirth, and cow survival in Pinzgauer, and 305-d fat yield, calving ease, and stillbirth in Tyrol Grey. Specifically, lactation milk, protein, and fat yields of Pinzgauer decreased by 55 kg (−1.05%), 2 kg (−1.18%), and 5 kg (−2.51%), respectively, in lactations of cows that had MB compared with single birth (*P* < 0.05). The fat yield of Tyrol Grey decreased by 3 kg (−1.55%) in lactations following MB compared with single birth (*P* < 0.05; Figure 2). Conflicting results regarding the effect of MB on milk production have been reported in the literature. For instance, Sawa et al. (2015) observed a significant increase in milk yield (+320 kg) in multiparous Polish Holsteins bearing twins compared with those bearing a single calf. Conversely, Schambow et al. (2021) reported that twin birth negatively affected the lactation milk yield of US Holstein cows (−142 kg). In the present study, Tyrol Grey experienced more calving difficulty in the presence of MB (score: 1.66) than single birth (score: 1.53, *P* < 0.05; Figure 2) in agreement with results of Gregory et al. (1996), who concluded that the requirement for assistance at calving for an experimental population that included several dairy, dual-purpose, and beef cattle breeds was more than twice as large in twin than single calvings (42.2% vs. 20.4%). Andreu-Vázquez et al. (2012) reported a need for assistance in 53.6% of twin calvings and 38.1% of singleton calvings in Spanish Holsteins. Generally, dystocia has a negative impact on subsequent milk production, particularly in early lactation, and taking into account that calving difficulty is more frequent in cows carrying twins, it is likely that the combination of MB and severe difficulty at calving leads to a reduction in dam performance. Stillbirth was much higher in the presence of MB than in single birth (Figure 2), both in Pinzgauer (10.40% vs. 2.95%; *P* < 0.05) and Tyrol Grey (7.52% vs. 1.25%; *P* < 0.05). Beerepoot et al. (1992) reported a mortality rate of 19% for twins and 5% for calves born from single calving within 24 h after birth. Hossein-Zadeh et al. (2008) defined stillbirth as the loss of calves from d 260 of gestation until calving and observed that 18.8% of twin births and 4.0% of single births were linked to stillbirth. Additionally, Strapáková et al. (2023) claimed that stillbirth is more common when MB results in 2 male calves than 2 females because males are heavier.

**Figure 2:**
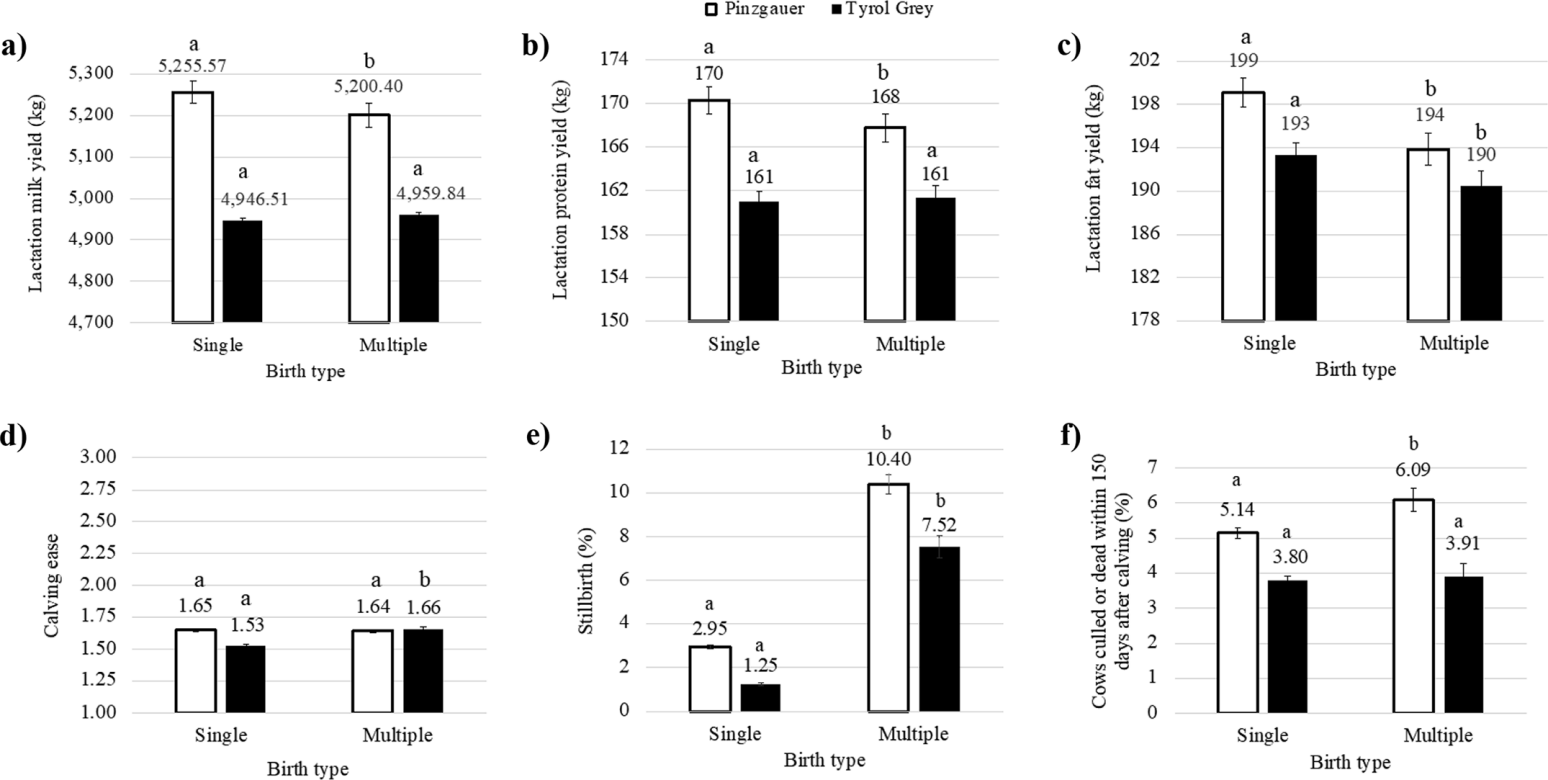
Least squares means of (a) milk, (b) protein, and (c) fat lactation yield, and (d) calving ease along with back-transformed LSM of (e) stillbirth and (f) cow survival (dam culled or dead within 150 d after calving) for the fixed effect of birth type. Values with different letters (a,b) within breed differ significantly (*P* < 0.05).

The percentage of Pinzgauer cows culled or dead within 150 d after calving was lower after single calvings (5.14%) than multiple calvings (6.09%, *P* < 0.05; Figure 2). The reasons for higher culling of cows after multiple calvings are often related to a higher incidence of postpartum issues (Cabrera and Fricke, 2021).

Variance components and h^2^ of MB were estimated separately for Pinzgauer and Tyrol Grey (Table 1). Although h^2^ was smaller in Pinzgauer than in Tyrol Grey (linear model: 0.029 and 0.040; threshold model: 0.138 and 0.200), the t was similar (linear model: 0.048 and 0.046). Threshold models are known to generate a greater h^2^ than linear models, but the 2 approaches are related to each other. Indeed, when we transformed the h^2^ on the liability scale to h^2^ on the observed scale (Dempster and Lerner, 1950), the estimates were 0.034 ± 0.004 and 0.038 ± 0.003 for Pinzgauer and Tyrol Grey, respectively. The Spearman's rank correlations between EBV from the linear and threshold models were >0.99 in both breeds. Widmer et al. (2022) estimated an h^2^ of 0.040 for Brown Swiss cows in Switzerland, which is in line with our findings. Weller et al. (2008) estimated h^2^ from 0.01 to 0.03 in Israeli Holsteins, depending on parity. Lower estimates were reported by Hossein-Zadeh et al. (2009) for first-parity Iranian Holsteins (h^2^ = 0.018), Moioli et al. (2017) for the Italian Maremmana breed (h^2^ = 0.014, t = 0.071), and Lett and Kirkpatrick (2018) for US Holsteins (h^2^ = 0.019, t = 0.044). By using data of various cattle breeds, Gregory et al. (1997) reported h^2^ up to 0.10. However, it is worth mentioning that the multibreed population investigated by these authors was selected for twinning on purpose within the framework of the twinning project of the US Meat Animal Research Center. In general, the magnitude of our h^2^ is comparable to that (approximately 0.020) of other functional traits in Austrian dual-purpose cattle breeds, such as the fertility index and rearing losses (Zuchtwert Austria, 2023).

**Table 1:** Additive genetic $\left(\sigma_{a}^{2}\right),$ permanent environmental $\left(\sigma_{pe}^{2}\right),$ herd-year $\left(\sigma_{h}^{2}\right),$ and residual variance $\left(\sigma_{e}^{2}\right)$ of multiple births along with the heritability (h^2^), with the SE provided for all the estimates

Model	Breed	$\sigma_{a}^{2}$	$\sigma_{pe}^{2}$	$\sigma_{h}^{2}$	$\sigma_{e}^{2}$	h^2^
Linear^1^	Pinzgauer	15.674 ± 1.816	10.703 ± 1.904	0.897 ± 0.741	522.247 ± 2.779	0.029 ± 0.003
	Tyrol Grey	14.708 ± 1.651	2.575 ± 1.439	3.379 ± 0.816	350.760 ± 2.240	0.040 ± 0.004
Threshold^2^	Pinzgauer	0.537 ± 0.0610	0.059 ± 0.0534	Negligible	Assumed^3^	0.138 ± 0.015
	Tyrol Grey	0.820 ± 0.0722	Negligible	Negligible	Assumed^3^	0.200 ± 0.014

1 Treated as binary: 0 (singleton) versus 100 (multiple birth).

2 Treated as binary: 0 (singleton) versus 1 (multiple birth).

3 The default logit link function adopted for the threshold model in ASReml holds the residual variance to π^2^/3 (Gilmour et al., 2015).

The present study reported that MB did not follow a specific trend in the dual-purpose breeds Pinzgauer and Tyrol Grey during the last 2 decades. In both breeds, we observed that MB negatively affected dam performance. Milk, fat, and protein yields of Pinzgauer following a MB were lower than yields following a singleton calving. In Tyrol Grey, only fat yield was significantly lower in cows that underwent MB compared with singleton calving. The MB increased calving difficulty in Tyrol Grey and resulted in higher stillbirth rates in both breeds. Considering that MB affects traits of economic relevance and that its additive genetic variance and h^2^ are different from zero, dedicated breeding programs would help to reduce MB occurrence or at least keep it stable in these 2 populations.
